# Deep phenotyping and genomic data from a nationally representative study on dementia in India

**DOI:** 10.1038/s41597-023-01941-6

**Published:** 2023-01-20

**Authors:** Jinkook Lee, Sarah Petrosyan, Pranali Khobragade, Joyita Banerjee, Sandy Chien, Bas Weerman, Alden Gross, Peifeng Hu, Jennifer A. Smith, Wei Zhao, Leon Aksman, Urvashi Jain, G. S. Shanthi, Ravi Kurup, Aruna Raman, Sankha Shubhra Chakrabarti, Indrajeet Singh Gambhir, Mathew Varghese, John P. John, Himanshu Joshi, Parvaiz A. Koul, Debabrata Goswami, Arunansu Talukdar, Rashmi Ranjan Mohanty, Y. Sathyanarayana Raju Yadati, Mekala Padmaja, Lalit Sankhe, Chhaya Rajguru, Monica Gupta, Govind Kumar, Minakshi Dhar, Jorge Jovicich, Andrea Ganna, Mary Ganguli, Prasun Chatterjee, Sunny Singhal, Rishav Bansal, Swati Bajpai, Gaurav Desai, Swaroop Bhatankar, Abhijith R. Rao, Palanimuthu T. Sivakumar, Krishna Prasad Muliyala, Preeti Sinha, Santosh Loganathan, Erik Meijer, Marco Angrisani, Jung Ki Kim, Sharmistha Dey, Perianayagam Arokiasamy, David E. Bloom, Arthur W. Toga, Sharon L. R. Kardia, Kenneth Langa, Eileen M. Crimmins, Aparajit B. Dey

**Affiliations:** 1 Center for Economic and Social Research, University of Southern California, Los Angeles, CA USA; 2 Department of Geriatric Medicine, All India Institute of Medical Sciences, New Delhi, India; 3 Department of Epidemiology, Johns Hopkins Bloomberg School of Public Health, Baltimore, Maryland USA; 4 Division of Geriatric Medicine, University of California, Los Angeles, Los Angeles, California USA; 5 Department of Epidemiology, School of Public Health, University of Michigan, Ann Arbor, MI USA; 6 Laboratory of Neuro Imaging, USC Stevens Neuroimaging and Informatics Institute, University of Southern California, Los Angeles, California USA; 7 Department of Economics, Finance and Real Estate, University of South Alabama, Mobile, USA; 8 Department of Geriatric Medicine, Madras Medical College, Chennai, India; 9 Department of Medicine, Government Medical College, Thiruvananthapuram, India; 10 Department of Geriatric Medicine, Institute of Medical Sciences, Banaras Hindu University, Varanasi, India; 11 Department of Psychiatry, National Institute of Mental Health and Neurosciences, Bengaluru, India; 12 Department of Internal and Pulmonary Medicine, Sher-e-Kashmir Institute of Medical Sciences, Srinagar, India; 13 Department of Medicine, Guwahati Medical College, Guwahati, India; 14 Department of Geriatric Medicine, Medical College, Kolkata, India; 15 Department of Medicine, All India Institute of Medical Sciences, Bhubaneshwar, India; 16 Department of Medicine, Nizam’s Institute of Medical Sciences, Hyderabad, India; 17 Department of Community Medicine, Grant Medical College and J.J. Hospital, Mumbai, India; 18 Department of General Medicine, Government Medical College and Hospital, Chandigarh, India; 19 Department of Medicine Indira Gandhi Institute of Medical Sciences, Patna, Bihar India; 20 Department of Medicine, All India Institute of Medical Sciences, Rishikesh, India; 21 Center for Mind/Brain Sciences, University of Trento, Rovereto, Italy; 22 Finnish Institute of Molecular Medicine, University of Helsinki, Helsinki, Finland; 23 Department of Psychiatry, University of Pittsburgh, Pittsburgh, PA USA; 24 Institute of Psychological Health, Mumbai, India; 25 School of Gerontology, University of Southern California, Los Angeles, CA USA; 26 Department of Biophysics, All India Institute of Medical Sciences, New Delhi, India; 27 Department of Development Studies, International Institute for Population Sciences, Mumbai, India; 28 Department of Global Health and Population, Harvard T.H. Chan School of Public Health, Boston, Massachusetts USA; 29 Department of Internal Medicine, University of Michigan, Ann Arbor, MI USA

**Keywords:** Risk factors, Predictive markers

## Abstract

The Harmonized Diagnostic Assessment of Dementia for the Longitudinal Aging Study in India (LASI-DAD) is a nationally representative in-depth study of cognitive aging and dementia. We present a publicly available dataset of harmonized cognitive measures of 4,096 adults 60 years of age and older in India, collected across 18 states and union territories. Blood samples were obtained to carry out whole blood and serum-based assays. Results are included in a venous blood specimen datafile that can be linked to the Harmonized LASI-DAD dataset. A global screening array of 960 LASI-DAD respondents is also publicly available for download, in addition to neuroimaging data on 137 LASI-DAD participants. Altogether, these datasets provide comprehensive information on older adults in India that allow researchers to further understand risk factors associated with cognitive impairment and dementia.

## Background & Summary

A rise in life expectancy is contributing to a rapid increase in the number of older individuals worldwide and is expected to lead to a sharp rise in Alzheimer’s disease (AD) from about 46 million people worldwide today, to more than 140 million in 2050^1^. The annual health care cost associated with AD is estimated to be over $1.1 trillion by 2050^2^. With a rising burden of all forms of dementia around the world, there is an urgent need to identify opportunities for preventing or delaying its onset.

The share of the Indian population aged 60 and above is projected to nearly double in the next 30 years from 10% in 2020 to 19% in 2050^3^. By 2050, the elderly population will reach around 300 million from the current 100 million^4^. In light of this rapidly increasing older population, the number of people with dementia and cognitive impairment is expected to rise as well. All prior dementia studies in India were based on geographically confined, selected communities within a single state. Even pooled estimates from meta-analyses of these prior studies fall short of representing the large, diverse population of India^5–7^.

In India, there exist significant regional differences in the sphere of longevity, health and disease burden, all of which might contribute to substantial geographic variability in the prevalence of dementia. For example, life expectancy at birth ranges from 64.8 years in Bihar to 75.1 years in Kerala^8^. According to India State-Level Disease Burden Initiative Collaborators^9^, diversity in the magnitude of disease burden and distributions of risk factors vary significantly across areas within the country. The critical need for a nationwide study of dementia that captures these diversities is widely recognized^5–7^.

We developed the first nationally-representative study of dementia in India, the Harmonized Diagnostic Assessment of Dementia for the Longitudinal Aging Study in India (LASI-DAD) with the aims of estimating the prevalence of dementia at the national level, investigating risk factors for dementia, assessing the burden of disease for Indian households, and examining cross-state variation in these outcomes of interest. LASI-DAD administered a rich battery of neuropsychological tests to adults aged 60 or older and interviewed their closest family member or caregiver. It drew its sample from a large-scale, nationally representative survey, the Longitudinal Aging Study in India (LASI). This has ensured national representativeness of the sample and availability of rich background information from LASI data. The LASI-DAD has adopted the Health and Retirement Study’s (HRS) Harmonized Cognitive Assessment Protocol (HCAP) to enable cross-country analysis, as well as additional cognitive tests drawn from 10/66 and other studies in India^10^. All cognitive tests were validated within the country before being used for the study.

## Methods

### Recruitment and sampling strategy

Harmonized Diagnostic Assessment of Dementia for LASI (LASI–DAD) drew a sub-sample of LASI respondents aged 60 and older (N = 4,096) in three phases from 2017 to 2020, on average 7 months after the main LASI interview. Drawn from the 2011 Census, the LASI sample is representative of both the country as a whole and each state and union territory. The LASI recruited older adults ages 45 and older and their spouses irrespective of age, with an oversample of individuals aged 65 and older. Its baseline survey was fielded in three phases from 2017 to 2019, interviewing 72,262 respondents from 42,726 households.

For the LASI-DAD, a two-stage stratified random sampling approach was adopted, oversampling individuals at high risk of cognitive impairment to ensure sufficient numbers of respondents with dementia or mild cognitive impairment. To accomplish this, we first classified respondents into those at high and low risk of cognitive impairment based on the core LASI study’s cognitive tests and on the proxy report for those who did not complete the cognitive tests. Specifically, we grouped the LASI respondents by age (60–69 and 70+) and education (no formal schooling vs. any education) and, within these groups, by relative performance on a battery of cognitive tests and proxy interviews. Respondents were classified to be at high risk of cognitive impairment if any of the following conditions were met: (1) overall cognitive test performance in the core LASI was in the bottom stratum-specific tertile; (2) memory score was below the 15th percentile; (3) non-memory cognitive scores were below the 15th percentile; (4) number of missing cognitive tests was above the 85th percentile; or (5) the IQCODE score from the proxy report was 3.9 or higher.

Once individuals were classified as having either low or high risk of cognitive impairment, we randomly drew an equal number of individuals from these two mutually exclusive groups within the sample quota predefined to ensure sex balance and the representation of the oldest old for each state^11^.

LASI-DAD included two interviews, one with the selected LASI respondent and one with an informant who was nominated by the respondent and believed to know them well. Similar to the main LASI study, for logistical reasons LASI-DAD was also fielded in three phases from 2017 to 2020. Based on the respondents’ preference, our interview team, which consisted of neuropsychology technicians, medical social workers and nurses, administered the HCAP protocol either at the hospital or the respondent’s home setting. All respondent interviews were conducted in-person. The informant interviews were conducted either face-to-face or by telephone. The field team travelled up to 12 hours by automobile to reach respondents residing in remote villages. The interview took an average of 170 minutes for the respondent interview (80 minutes for cognitive testing and 90 minutes for geriatric assessment) and 22 minutes for the informant interview.

### LASI-DAD protocol

Our study protocol also includes venous blood specimen (VBS) collection and assays. Certified and trained phlebotomists from our industry partner, Metropolis laboratory, drew 17 ml of VBS from respondents as the first part of the cognitive interview and preferably fasting blood sample which was noted in the questionnaire. Four of the five tubes were processed within an hour, yielding whole blood (for Complete Blood Count and HbA1c assay), serum, plasma, and buffy coat. All specimens were shipped to the Metropolis laboratory in Delhi within approximately 24 hours via a cold chain (−20 °C for plasma and 4 °C for other specimens) where lab work was completed for 33 assays (see Table 2 for the complete list of assays). Our genomics initiative partner, MedGenome, picked up the remaining tubes and shipped them directly to their laboratory based in Bangalore for DNA extraction.

Out of a total of 5,074 selected individuals, 4,096 completed the LASI-DAD interview with a final response rate of 80.7%. Table 1 presents the sample characteristics of the LASI-DAD, together with that of the LASI. There were 30 cases where only an informant interview was conducted as the LASI-DAD respondent was unable to complete the interview. Respondents were unable to complete the interview if they were bedridden, unable to speak, or were experiencing psychiatric disorders that hindered them from participating. There were 49 cases of respondent-only interviews since an informant interview could not be completed in these cases. Altogether, 98.8% of cases included both a respondent and informant interview, and 2,844 participants consented to give venous blood samples, leading to a response rate of 69.4% for blood draws. Among the 19.3% nonresponse to the LASI-DAD interview, 6.0% refused to respond, 5.3% were due to inability of interviewers to contact sample residents, and 8.0% were attributable to other reasons (e.g., the resident was willing to be interviewed but could not do so for health-related reasons). About 2.1% of the selected participants were deceased, and therefore could not be interviewed.

**Table 1:** Characteristics of LASI and LASI-DAD Sample^a^.

	LASI, Age 60+		LASI-DAD	
	N	%	N	%
Age				
60–64	10 134	29.9	1291	30.9
65–69	8845	28.6	1179	30.9
70–74	5746	18.8	720	17
75+	6752	22.7	906	21.3
Sex				
Male	15 106	47.5	1889	49.2
Female	16 371	52.5	2207	50.8
Education				
None	16 894	56.5	2009	55.7
Primary school or less	7562	22.6	1076	23.3
Middle to secondary	4616	13.9	695	14.3
Higher secondary+	2405	7	316	6.7
Urbanicity				
Urban	10 747	29.5	1557	29.4
Rural	20 730	70.5	2539	70.6
Total	31 477	100	4096	100

^a^Sample size (N) is unweighted. % represents weighted distribution.

The DAD protocol consisted of the following (Table 2 presents the detailed protocol):

**Table 2:** Longitudinal Aging Study in India-Diagnostic Assessment of Dementia Protocol.

Respondent Interview		
Cognitive Tests (N = 4096)		Geriatric Assessment (N = 4084)
Hindi-Mental State Examination		Blood pressure
Naming objects, prime Minister		Pulse
Word List Learning – Immediate, delayed, recognition		Anthropometry
Digit Span Forward and Backward		Activities of Daily Living (ADL)
Symbol Cancellation Test		Instrumental Activities of Daily Living (IADL)
Story recall – Immediate, delayed, recognition		Anxiety
Constructional Praxis – Copy, delayed		Depression
Semantic Fluency (Animal Naming Test)		Timed Up and Go Test (N = 774)^b^
Hand Movement Sequence Test (N = 2279)^a^		6-Minute Walk Test (N = 359)^b^
Token Test (N = 2233)^a^		Mini Nutritional Assessment
Judgment, Problem Solving (N = 2295)^a^		Hearing test
Serial 7 s		Medicine Use (N = 2496)^a^
Community Screening Instrument for Dementia		
Raven’s Standard Progressive Matrices		
Go-No Go Test		
**Informant Interview** (N = 4029)		
Informant’s relationship with respondent		
Informant Questionnaire on Cognitive Decline in the Elderly		
Blessed Dementia Rating Scale		
Community Screening Interview - Dementia Cognitive		
Daily Activities Questionnaire		
10/66 Dementia Research Group Informant Questionnaire		
**VENOUS BLOOD ASSAYS** (N = 2892)		
**Complete blood cell counts**	LDL/HDL Ratio	Blood Urea Nitrogen
Hemoglobin	Chol/HDL Ratio	Creatinine
Red Blood Cell Count	Triglycerides	Uric acid
Red Cell Distribution Width	**Liver Function Tests**	Calcium
Differential Leucocyte Count	Bilirubin (Total)	**Thyroid Function Tests**
Mean Corpuscular Hemoglobin	Bilirubin Direct	Total Thyroxine
Concentration	Bilirubin Indirect	Total Triiodothyronine
Mean Corpuscular Hemoglobin	Total Protein	Thyroid stimulating hormone
Mean Corpuscular Volume	Albumin	(TSH)
Packed Cell Volume	Globulin	**Other tests**
Glycosylated hemoglobin (HbA1c)	A/G Ratio	Vitamin B12
**Serum Based Tests**	Alanine Aminotransferase	Folic acid
Glucose	Aspartate Aminotransferase	25-hydroxy vitamin D
**Lipid Profile**	Alkaline Phosphatase	Homocysteine
Total Cholesterol	Gamma-Glutamyl Transferase	NT pro BNP
HDL Cholesterol	**Renal Function Tests and**	High-sensitivity C-Reactive Protein (hsCRP)
LDL Cholesterol	**Electrolytes**	Lipoprotein (a)
VLDL Cholesterol		

^a^Protocol added in Phase 2.; ^b^Only administered in hospital setting.

#### Cognitive tests

Neurocognitive testing was conducted to measure different domains of cognition including memory, constructional praxis, executive function, processing speed, and language. All tests were validated in India. The instrument was translated into 12 languages for the dementia study: Hindi, Kannada, Malayalam, Gujarati, Tamil, Punjabi, Urdu, Bengali, Assamese, Odiya, Marathi, and Telugu. To ensure accuracy, we first translated the tests into local languages, then independent research staff, fluent in both languages, back-translated the tests^12^. Any inconsistencies were resolved by consulting with local language experts. Additional cognitive tests were added in the second phase to better measure the domains of executive functioning, judgment, and problem-solving capability.

#### Informant report

An interview of the relative/friend about the respondent’s cognition and daily activities.

#### Geriatric assessment

A geriatric interview was conducted to measure physiological markers of aging. The interview consists of specific physical biomarkers including blood pressure, height, weight, mid-arm circumference, calf circumference, knee height, objective tests of functionality (Timed up and go test, Six-minute walk test) and questions on activities of daily living (ADLs), nutrition, anxiety, depression and a hearing test. Timed get up and go test and Six-minute walk tests were administered only at hospital settings due to space limitations at residential settings.

#### Venous blood sample

A blood sample was collected to carry out a range of whole blood and serum-based assays (see Table 2 for a complete list of assays and Supplementary Table 1 for descriptive results of each assay). The blood sample was also used to extract DNA for the Genomics study.

#### Neuroimaging

We conducted a pilot neuroimaging study in a subsample of 137 LASI-DAD participants. The MRI protocol is based on that developed in ADNI-3 (Alzheimer’s Disease Neuroimaging Initiative; http://adni.loni.usc.edu) for the Philips Ingenia 3 T and Siemens Skyra 3 T scanners at three sites: Bangalore, Mumbai, and Kolkata. The 55-minute scanning protocol included structural two 3D T1, 3D FLAIR, high-resolution 2D T2 of the hippocampus, resting-state functional MRI, diffusion MRI, and susceptibility weighted MRI. All scanners used a 32-channel head RF receive coil. Two structural 3D T1-weighted images were acquired in sagittal orientation using a three-dimensional magnetization prepared rapid acquisition gradient echo sequence (3D MP-RAGE; TR = 2300 ms; TE = 2.03 ms; flip angle = 8 degrees; voxel size = 1x1x1 mm3; FOV = 24 cm; slice thickness = 1 mm; GRAPPA 2 acceleration), one at the beginning and one at the end of the MRI acquisition protocol. Two acquisitions were acquired because it has been seen that averaging them increases reliability in the automated cortical parcellation and subcortical segmentation^13^. A 3D T2-weighted fluid-attenuated inversion recovery (FLAIR; TE = 221 ms; TR = 4800 ms; voxel size = 1x1x1 mm3; slice thickness = 1 mm; GRAPPA 2 acceleration) scan was acquired to visualize white matter hyperintensities, which are indicative of cerebrovascular pathology and to improve pial segmentation. A high-resolution 2D T2-weighted sequence (TR = 3844 ms; TE = 120 ms; flip angle = 90 degrees; voxel size = 0.45 × 0.45 × 2 mm2; slice thickness = 2 mm) was acquired perpendicular to the long axis of the hippocampus to help the automated segmentation of the hippocampal subfields^14,15^. Resting state functional MRI was acquired with TR = 8400 ms, TE = 85 ms, flip angle = 90 degrees, voxel size = 3 × 3 × 3.424 mm3, slice thickness = 3.2 mm. Diffusion imaging was acquired with a single b = 1000 s/mm2 shell with TR = 8020 ms, TE = 56 ms and flip angle = 122 degrees.

#### Clinical dementia rating

With the aim of bringing together expert clinical judgement for the diagnosis of dementia, we developed an online clinical consensus panel approach for our study. The online platform provides standardized data necessary for clinicians to rate respondents on the Clinical Dementia Rating (CDR®)^16^_,_ and its validity was demonstrated by its comparison to in-person clinical assessment and consensus diagnosis^17^_._ This approach is a cost-effective alternative to the typical in-person clinical diagnosis consensus conference.

### Genotyping

DNA extraction and genotyping were performed by MedGenome (Bangalore, Karnataka, India). A total of 960 LASI-DAD participants were genotyped using the Illumina Infinium Global Screening Array-24 (GSA) BeadChip, version 2.0 (Illumina, San Diego, CA). All the raw imaging files were released to the Quality Assurance/Quality Control (QA/QC) analysis team at the University of Michigan. The initial genotyping calling was performed in GenomeStudio version 2.0, with Genotyping Module version 2.0.4 and GenTrain version 3.0. Auto-clustering was used to define the cluster boundaries, and the “GSA-24v2-0_A1” annotation file (genome build 37/hg 19) was used to annotate SNPs. QC procedures are described below. Among the 665,608 probes that were present on the array, 533,348 polymorphic probes (80.1%) were available to be used for imputation after QC. Imputation to the 1000 G Genomes Project reference panel phase 3 version 5 (initial release on May 2013, haplotypes released Oct 2014) was performed using the Michigan Imputation Server, Minimac 4, version 1.0 (Center for Statistical Genetics, University of Michigan, Ann Arbor, MI) with phasing performed using Eagle2.4. Imputation to the Trans-Omics for Precision Medicine (TOPMed) reference panel (r2) was performed using the TOPMed Imputation Server with the same algorithm.

### Polygenic risk score construction

To capture genetic risk for Alzheimer’s disease and impairment in later-life cognitive function, we created polygenic risk scores (PRSs)^18^ using summary statistics from four published GWAS meta-analyses: three meta-analyses of Alzheimer’s disease^19–21^ and one meta-analysis of general cognitive function^22^. For all meta-analyses, we constructed PRSs using genome-wide significant SNPs only (P < 5 × 10^−8^), denoted as “top SNPs” PRSs. In addition, for general cognitive function, we also generated a PRS for all independent SNPs with (P < 1 × 10^−4^) after clumping (<0.25 within a 250 kb window) using the LD structure in South Asian ancestry from the 1000 Genomes reference panel, denoted as an “all SNPs” PRS. In either case, only SNPs with high imputation quality (R^2^ > 0.8) in LASI-DAD were included. PRSs were coded such that higher PRS represented increased risk for Alzheimer’s disease or higher general cognitive function. For more information on the LASI-DAD polygenic risk scores, see the PRS – Release 1 report available at the LASI-DAD website (https://lasi-dad.org/dpInstrument).

### Consensus clinical dementia rating (CDR®)

A web-based approach to diagnostic consensus presents a promising way to achieve experts’ review, analysis, and consensus at much lower costs. LASI-DAD used the Clinical Dementia Rating (CDR®)^16^ for the basis of the clinical diagnosis of dementia. The CDR® is a global rating device that was first introduced in a prospective study of patients with dementia^23^ and is now one of the most widely used measures^24,25^.

The CDR® is comprised of six cognitive and functional domains: (1) memory; (2) orientation; (3) judgment and problem solving; (4) community affairs; (5) home and hobbies; and (6) personal care^16^. Clinicians were asked to complete the CDR® ratings based on cognitive test results and informant reports. The scales for the first five domains are a 5-point ordinal scale: 0 = no impairment, 0.5 = questionable impairment, 1 = mild impairment, 2 = moderate impairment, and 3 = severe impairment. The personal care domain is scored on a 4-point scale with possible scores of 0, 1, 2, and 3. We recruited clinicians with CDR® training (http://alzheimer.wustl.edu/cdr/cdr.htm), and CDR®- certified expert clinicians were presented with the following information:

1. basic background information, such as age, marital status, sex, education in completed years, occupation, language, self-rated memory, memory compared to two years ago, and date last examined by the LASI-DAD interviewer;
2. results from the cognitive tests extracted from the LASI-DAD, including summary information from the HMSE, TICS, CSI-D, judgement & problem solving, numeracy, ADL, IADL, mobility, CESD, and anxiety;
3. information obtained from the informant interview, such as informant-related questions (relationship with the respondent, whether informant is a caregiver, the number of years known respondent, and the frequency the informant has seen the respondent in the past year), respondent’s mental status (IQCODE, 10–66, and CSI-D results), respondents’ activities, and interviewer observations; and
4. relevant health history information collected from both the LASI-DAD geriatric assessment and the core LASI interview on blood pressure (systolic and diastolic), stroke, heart disease, diabetes, hypertension, depression, Alzheimer’s disease/dementia, psychiatric problems, neurological problems, vision impairment, hearing impairment, and incontinence.

Based on this information, each clinician provided domain-specific ratings and an overall summary CDR® score was calculated based on an algorithm that uses these ratings. Each case was assigned to three CDR®-certified clinicians, who were asked to review cases independently. If there were inconsistent ratings, clinicians were asked to engage with each other virtually in order to review each other’s comments and resolve inconsistencies. While consensus was reached in many cases throughout this process, there were still some that needed further action. For the cases where consensus could not be reached, an online consensus meeting was organized and hosted by a moderator in order to further discuss and reach a consensus on a final CDR® score. Clinicians who evaluated the case initially were not always present at the consensus call, and all participating clinicians in the call review and discuss each case. Moderators were asked to mark whether a consensus was reached through discussion (see Lee *et al*.^26^ for further details on the online clinical consensus diagnosis). Finally, for Wave 1 of LASI-DAD, clinical consensus ratings were obtained for cases in Phases 2 and 3 only.

## Data Records

The Harmonized LASI-DAD data and the Venous Blood Assay data file and documentation are available from the Gateway to Global Aging Website in STATA format^27^. The LASI-DAD Global Screening Array data is available for download through The National Institute on Aging Genetics of Alzheimer’s Disease Data Storage Site (NIAGADS) (https://dss.niagads.org/datasets/ng00106/). The LASI-DAD neuroimaging data are available through LONI ADI (https://ida.loni.usc.edu/) project name ‘DAD’_._

### Harmonized longitudinal aging study in india – diagnostic assessment of dementia (LASI-DAD)

The Harmonized LASI-DAD data are contained in a single file and are stored in a “fat format”, where rows correspond to individuals and columns to variables. The unit of observation is the individual. Each individual is uniquely identified by the identifier “PRIM_KEY”. Households are identified by “HHID”. The variable names in this dataset follow a consistent pattern. The first character indicates whether the variable refers to the references person (“R”) or the household (“H”). The second character indicates the wave to which the variable pertains: “1” (as new waves of data will be collected, the wave indicator will take values 1 for the first wave, 2 for the second wave, and so on) or “A”. The “A” indicates “all”, which is used when the variable is not specific to a single wave. For example, RABYEAR, the birth year of the respondent. The remaining characters describe the concept that the variable captures.

The Harmonized LASI-DAD includes data on the respondent’s demographic characteristics, cognition, informant report, health and physical measures, polygenic risk scores, and consensus clinical dementia rating. This data file also includes sample weights meant to account for differential selection probabilities produced by the adopted sampling strategy, and to adjust for differential non-response across demographic groups. Specifically, sample weights allow one to align the LASI-DAD sample distributions of gender, age, literacy, and urbanicity to the corresponding distributions in the Indian population age 60 and older.

### Venous blood specimen

The venous blood specimen (VBS) datafile contains results from the VBS assays conducted by Metropolis laboratory in India. A complete list of assays can be found in Table 2. This datafile can be linked to the Harmonized LASI-DAD dataset using the unique individual identifier “PRIM_KEY”. Descriptive results of the bioassays can be found in Supplementary Table 1. The sample size of the bioassays varies slightly due to insufficient serum quantity for some tests or unreliable values resulting from questionable quality of whole blood specimens.

### Global screening array

In 2018, 960 respondents from LASI-DAD who consented to the blood sample collection were genotyped using the Illumina Infinium Global Screening Array-24 v2.0 BeadChip (GSA array). Three datasets are publicly available for download at NIAGADS (https://dss.niagads.org/datasets/ng00106/). The first dataset (accession number fsa000011) is the original genotype data from the GSA array, containing 1008 scans derived from 993 unique subjects (including 960 LASI-DAD subjects and 33 control subjects from the 1000 Genomes Project). The second dataset (accession number fsa000012) contains genotype data imputed to the 1000 G reference panel (phase 3 v5). The third dataset (accession number fsa000013) contains genotype data imputed to the TOPMed reference panel (r2). The second and third datasets contain 960 unique LASI-DAD subjects and are in VCF format.

### Neuroimaging data

Neuroimaging data are available through the Image and Data Archive (IDA) online database hosted by the Laboratory of Neuro Imaging (LONI) at the University of Southern California (https://adni.loni.usc.edu/). All available MRI modalities (described in the Methods) are available for download in DICOM or NIFTI file formats. Currently the LASI-DAD neuroimaging data are hosted under a restricted-sharing policy: permission can be requested by submitting a data use application.

## Technical Validation

### Cognitive test validity

Cognitive tests were evaluated through two pretests^28^. The first pretest was carried out in November 2016 in New Delhi, Tamil Nadu, and Uttar Pradesh, and the second in February 2017 in Kerala, Karnataka, and Rajasthan. Adjustments were made to the protocol based on the observations and feedback received from these two pretests. For example, the HRS-HRCAP CERAD (https://agingcenter.duke.edu/cerad) word list memory and recall test was difficult to adapt due to a significant share of the respondents being illiterate and respondents’ visual impairment due to advanced age. The test was therefore modified as follows. The interviewer read each word to the respondent, who was asked to repeat each word aloud. The respondent was then tested on immediate recall.

Furthermore, some tests were omitted to avoid redundancy. The Montreal Cognitive Assessment test (MOCA)^29^ was initially included, but was taken out after the first pretest. Many items in the MOCA were repetitions of other cognition tests like the Hindi Mental State Exam (HMSE) and the Telephone Interview for Cognitive Status (TICS). Additionally, some questions in MOCA like the animal-naming tests had pictures of animals like rhinoceros, which the majority of participants could not answer as this animal is not commonly seen in India. Similarly, we administered both the black-and-white Raven’s test^30^, which is a part of the HRS–HCAP study, and a Color Matrix Progression test, which is considered to be easier and to work better among illiterate populations. From the phase 1 data, we observed respondents’ fatigue when we administered two versions of the Raven’s test one after another. Specifically, respondents performed worse on the colored version, which was administered after the black-and-white version. Therefore, we kept only the black-and-white version of the Raven’s test.

In addition to the HRS–HCAP cognitive test batteries, we added a digit span forward before we administered a digit backward test^31^. The test helps study the attention and psychomotor speed domain of cognition.

**Fig. 1:**
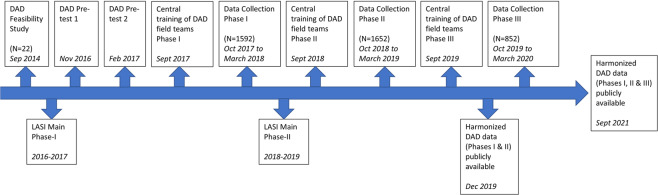
Timeline of fieldwork for LASI-DAD.

After the two pretests, the cognitive test protocol was finalized for the main data collection, which started in October 2017. A summary of the fieldwork timeline is presented in Fig. 1. The selected tests (see Table 1) cover the essential domains required to diagnose major neurocognitive disorder or dementia as stated in the *Diagnostic and Statistical Manual of Mental Disorders* or DSM-5 guidelines^32^, namely complex attention (symbol cancellation, digit span, and Go–No Go test), executive function (Raven’s matrices, clock drawing), learning and memory (CERAD word recall, logical memory), language (object naming, animal naming, Community Screening Interview for Dementia), and perceptual motor function (constructional praxis).

Backward counting from 100 was tested during the data collection in phase-1. The interviewers had difficulty counting the number of errors the participant made, as the errors could range from missing 1–2 numbers to skipping as many as 10 numbers. Proper scoring of this test turned out to be very difficult. Despite considerable efforts by the neuropsychologist to develop a consistent scoring rule, scoring continued to be an issue, and the test was subsequently dropped in the second phase of the study. In the second phase of data collection, we added the executive function and the judgment and problem-solving questions, as these domains were underrepresented in the questionnaire. The additional tests were the hand-movement sequence^33^ and the Token test^34^ for executive function.

We conducted a confirmatory factor analysis of the full battery of cognitive tests that identified salient domains consistent with a priori theory, evaluating intercorrelations among tests and demonstrating construct validity^35^.

### Effort to ensure bioassay data validity

For bioassays, the LASI-DAD team collaborated with Metropolis Laboratory, the leading independent pathology laboratory in India that offers a comprehensive menu of over 4,500 tests in clinical chemistry, clinical microbiology, cytogenetics, hematology, molecular diagnostics, and surgical pathology. Metropolis delivers over 30 million tests a year, catering to more than 10,000 hospitals, nursing homes, and other laboratories. It is accredited by NABL (National Accreditation Board for Testing and Calibration Laboratories) in India and has performed consistently well in all external quality control programs.

For assay quality control (QC), Metropolis laboratory in Delhi runs QC samples every morning before testing clinical and LASI-DAD study samples. The number of QC samples measured varies from two (low or high levels) to three (low, mid, or high levels), depending on the assay. The laboratory protocol requires that testing of clinical/study samples would not be initiated if the value of one QC sample is beyond three standard deviations (S.D.) from the laboratory-established mean or values of two QC samples are beyond two S.D. from the mean. During the period when the LASI-DAD study samples were tested, the LASI-DAD team also independently monitored QC sample results on real-time basis. For all assays, QC sample values were within the criteria established by Metropolis laboratory.

LASI-DAD also participated in the effort to cross-calibrate total and HDL cholesterol, glycosylated hemoglobin (HbA1c), and C-reactive protein (CRP) data across laboratories for large community-based aging surveys in several countries, mostly within the family of Health and Retirement Studies (HRS). Duplicate samples were prepared, shipped via a cold chain, and tested at Metropolis laboratory and at the reference laboratory at University of Washington, Seattle, WA. The R-squares for the linear regression of the assay values from these two laboratories were 1.00 for total cholesterol, 0.99 for HDL cholesterol, 1.00 for HbA1c, and 0.96 for CRP.

### DNA quality

DNA was extracted using QIAsymphony DNA Midi Kit (Cat No. 931255) on a QIAsymphony (QIAGEN, Germany) instrument as per the manufacturer instructions. After extraction, DNA samples were subjected to Qubit for quantifying the DNA and QIAXPERT for 260/280 nm purity ratio. Further, the integrity of the DNA was assessed by agarose gel electrophoresis.

### Genotyping quality

In addition to the LASI-DAD samples, a total of 38 samples from the 1000 Genomes Project (1000 G), including 33 unique subjects, were put into genotyping production as external controls. The samples were genotyped in batches corresponding to 96-well plates. Each plate contained two to five 1000 G controls, as well as an average of two study sample duplicates across five plates. Genotype quality control included a sex check, a relatedness check, and population structure analysis. Samples with a missing call rate greater than 2% were removed. Further, we removed SNPs that met any of the following criteria: SNPs with a missing call rate greater than 2%, greater than 0 discordant calls among 15 pairs of technical duplicates, greater than 0 discordant calls among 20 1000 G control samples, sex differences in allele frequency greater than 0.2, sex differences in heterozygosity greater than 0.3, more than one Mendelian error in 1000 G controls, and any positional duplicates. Table 3 shows a summary of SNP genotyping failures and missingness by chromosome type. Table 4 shows the number of SNPs with Mendelian errors. Figure 2 shows a summary of concordance by SNP over 15 duplicate sample pairs, binned by minor allele frequency. For additional information, request access to the LASI QC report, available at NIAGADS (https://dss.niagads.org/datasets/ng00106/).

**Table 3:** Summary of SNP genotyping failures and missingness by chromosome type.

	A	M	U	X	Y	XY
Number of probes	629637	1180	4	28594	4964	1229
SNP tech failures	0.0042	0.0017	1	0.0065	0.0189	0.0008
Missing >0.05	0.0005	0.0119	0	0.0051	0.0054	0

A = autosomal, M = mitochondrial, U = unknown position, X = X chromosome, XY = pseudoautosomal, Y = Y chromosome.

The row “SNP tech” gives the fraction of SNPs that failed preliminary QC. The row “Missing > 0.05” gives the fraction of SNPs that passed preliminary QC and that have a missing call rate (*missing.n1*) >0.05.

**Table 4:** Number of SNPs with Mendelian errors.

# Mendelian errors	# SNPs
0	2386
1	37
2	7
3	2
4	2
5	0

**Fig. 2:**
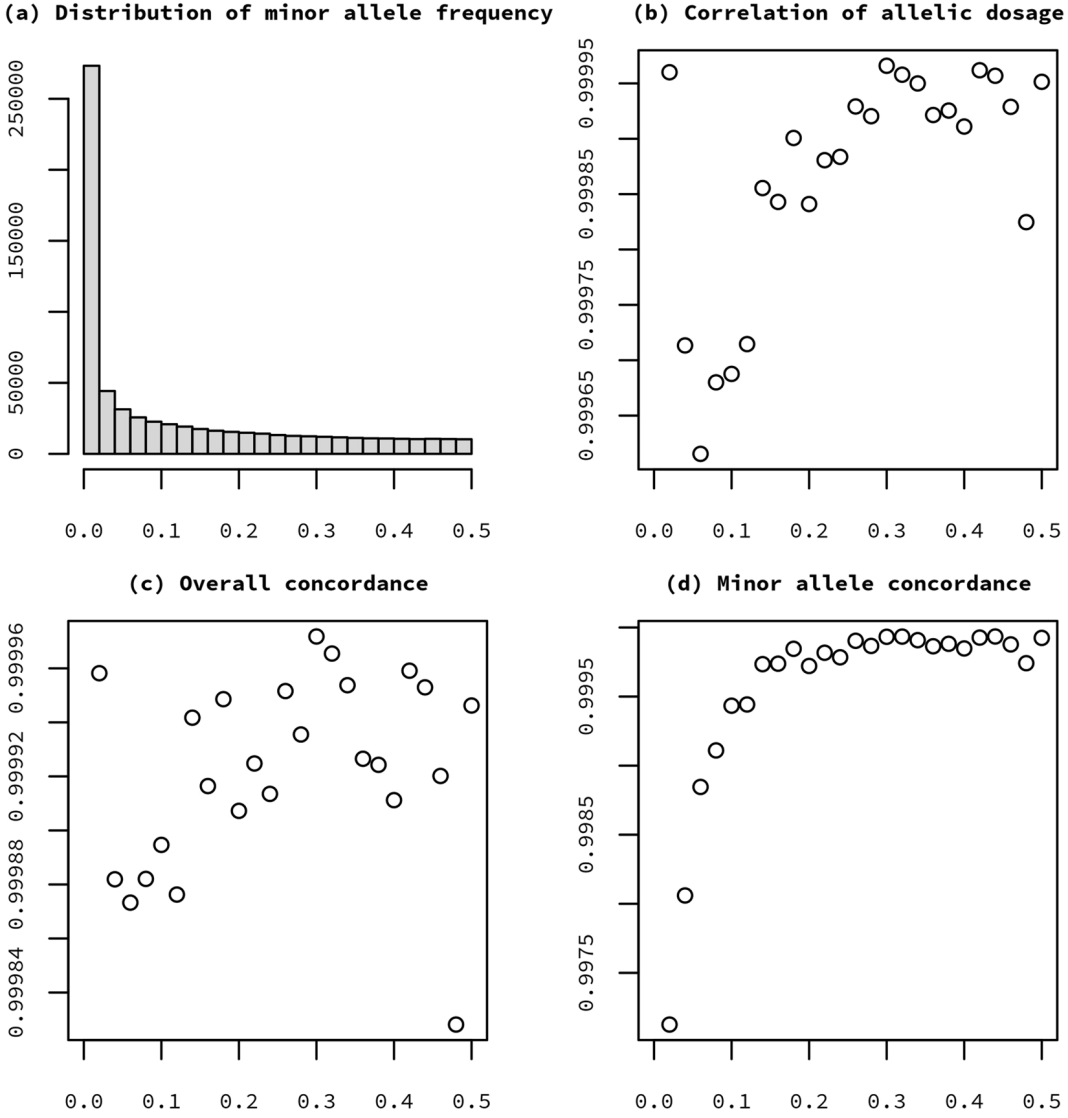
Summary of concordance by SNP over 15 duplicate sample pairs, binned by minor allele frequency. (**a**) Distribution of minor allele frequency, b) mean of correlation of allelic dosage, (**c**) mean of concordance, (**d**) mean of minor allele concordance.

### Imputation quality

**Fig. 3:**
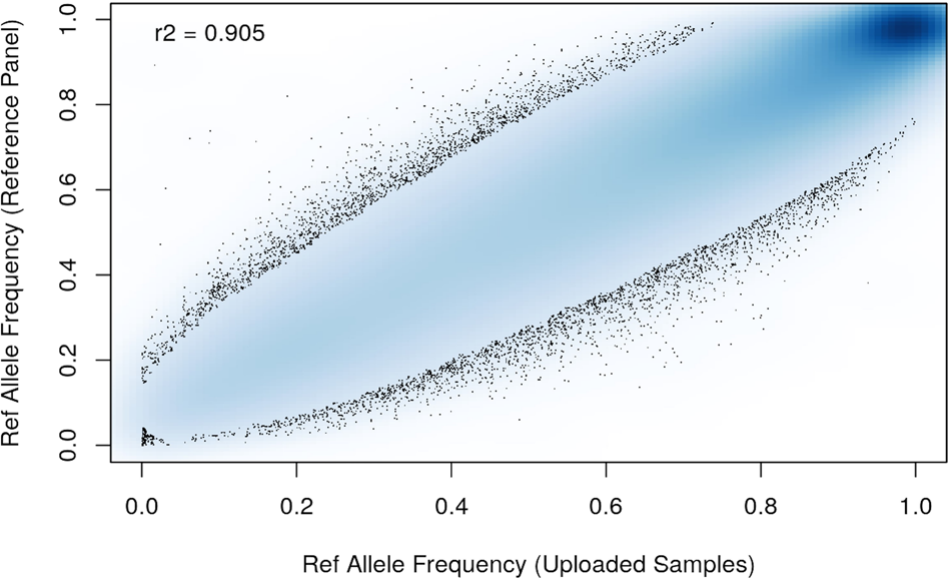
Allele frequency check for comparing study (x-axis) and reference panel (y-axis) alternate allele frequencies. The figure shows the imputation basis SNPs on all autosomes and X chromosome. All autosomes and X chromosome were submitted for the full set of 960 samples (i.e. no whole chromosome anomaly exclusions).

For both the 1000 G and TOPMed imputed data, we masked known SNP genotypes during imputation and compared the imputed vs. known genotypes to determine concordance (empirical dosage r^2^). Mean empirical dosage r^2^ for the 1000 G imputed data was 0.857 for SNPs with minor allele frequency (MAF) ≥ 0.05 and 0.657 for SNPs/variants with MAF < 0.05. Mean empirical dosage r^2^ for the TOPMed imputed data was 0.861 for SNPs with minor allele frequency (MAF) ≥ 0.05 and 0.662 for SNPs/variants with MAF < 0.05. Quality metrics for all masked SNPs (genotyped SNPs in the imputation output), dichotomized into groups of MAF ≥ 0.05 vs. MAF < 0.05 are shown in Table 5. The allele frequency check for comparing study and reference panel alternate allele frequencies is shown in Fig. 3. For additional information, request access to the LASI 1000 G and TOPMed imputation reports, available at NIAGADS (https://dss.niagads.org/datasets/ng00106/).

**Table 5:** Quality metrics for all masked SNPs (genotyped SNPs in the imputation output), dichotomized into groups of MAF ≥0.05 vs. MAF <0.05.

MAF (in study samples)	Number of markers	Metric	EmpRsq	EmpR	Dose0
<0.05	200317	mean	0.662	0.788	0.807
<0.05	200317	median	0.713	0.844	0.875
≥0.05	307991	mean	0.861	0.923	0.911
≥0.05	307991	median	0.917	0.958	0.953

The second column shows the number of markers in each MAF group. Mean and median values are presented for empirical dosage r^2^ (EmpRsq in Minimac4 results, comparing imputed and true genotypes) and the Dose0 metric. No LooRsq threshold has been applied here, such that all masked and imputed SNPs in each MAF category are included in these averages.

## Usage Notes

Ethics approval was obtained by the University of Southern California Institutional Review Board (UP15-00684) and the Indian Council of Medical Research for the All India Institute of Medical Science (54/01/Indo-foreign/Ger/16-NCD-II).

The Harmonized LASI-DAD data file can be linked to the Harmonized LASI dataset using the unique individual identifier “PRIM_KEY”. The Harmonized LASI-DAD dataset also contains the variable “R1LASIDY” to indicate the number of days between the LASI and DAD interview. By linking the Harmonized LASI-DAD and Harmonized LASI datasets, the information can be used to increase our knowledge on the determinants of late-life cognition, cognitive aging, and dementia.

The LASI-DAD has several important strengths. First, LASI-DAD provides nationally representative data that enable inferences to be made about the cognition of the 60-year and older Indian population. LASI-DAD provides important information about cognitive functioning as well as informant reports. Despite the considerable interview administration time, response rates are relatively high, thereby reducing sample selectivity concerns. The LASI-DAD instruments also cover all domains essential for clinical dementia rating in the elderly Indian population, which is largely illiterate. We adapted and modified the test to best suit the population. The instrument was translated into twelve different languages, and all tests were pretested multiple times, ensuring their validity in the local context. The protocol includes a range of cognitive tests that work best for an illiterate population. It also incorporates different neuropsychological tests to measure the same domains of cognition, providing robust domain-specific cognition measures. Furthermore, the resulting data can be harmonized with the HRS-HCAP surveys and other sister studies, thereby permitting international comparisons of the findings in LASI-DAD with those in other studies.

There are also several methodological innovations. We adopted Computer Assisted Personal Interviewing (CAPI) for the administration of cognitive tests and geriatric assessments to respondents and informant interviews. Collected data were uploaded to the server on a daily basis, allowing the data management team to review the collected information in real-time. This setup enables the core management team to monitor the quality of the data collected and to raise concerns or questions in near real time. Our operations team also developed a venous blood specimen (VBS) management protocol, tracking VBS through collection, handling, shipment, and assaying. The system allows the recording and monitoring of the shipping times and temperature during the transit period, as the literature has shown that both shipping duration and temperature potentially either increase or decrease the results reported by the laboratory.

Another important strength of the project is its rich data on risk factors, including both genotype and phenotype data, enabling all interested researchers around the world to further study and improve the current understanding of Alzheimer’s disease and dementia. For a sub-sample, we completed the Global Screening Array using the Illumina Infinium GSA-24 v2.0 Beadchip. Through a geriatric clinical assessment, we collected data on anthropometry, blood pressure, questions on functional status, such as basic and instrumental activities of daily living, depression, anxiety, nutrition, as well as VBS assays. Furthermore, we are able to link the data to the main LASI survey, which includes a comprehensive information on socioeconomic status, medical history, health behaviours, including smoking and alcohol use, and other demographic information.

While the LASI-DAD study provides rich, population-representative data on late-life cognition and dementia, several limitations are worth acknowledging. The study sample was not drawn from all the states of India, as it recruited the sample from 18 states and union territories. It was also difficult to locate respondents in some areas, such as slums and remote villages, and sending a phlebotomist to such areas was problematic. The fear associated with giving blood samples and the 17 ml blood sample amount has led to a 69% response rate for venous blood collection. Furthermore, due to the COVID-19 pandemic, fieldwork was stopped earlier than planned. This prevented the research team from obtaining the target sample size in Madhya Pradesh and Maharashtra.

In preparation for Wave 2 of LASI-DAD, the study team conducted two pilot studies to test new components of the instruments (e.g., an additional cognitive test like the trail-making test, informant questions regarding judgement and problem-solving capacity and caregiving burden) and examine the feasibility of fieldwork operations for the food frequency questionnaire, audiometry, and retinography. The first pilot study was conducted in September 2021. The instruments were revised based on feedback from the field team. The retinography protocol was dropped due to difficulty associated with administration in the field. The second pilot was conducted from May 24 to July 5, 2022 at three study sites: Delhi, Jaipur, and Pondicherry. During this pilot, the study team administered the entire Wave 2 protocol.

The LASI-DAD study team is currently in the field conducting Wave 2 interviews. We aim to conduct follow-up interviews with all Wave 1 respondents (N = 4,096). If the respondents are deceased, we conduct an end-of-life interview with an informant to understand the cognitive state of the respondent before they died and the nature of their passing. We also plan to recruit about 840 newly age-eligible respondents who are 60–64 years old and 840 new respondents older than 64, as we are expecting a high mortality due to the COVID-19 pandemic. For all new respondents, we follow the sample sampling strategy we used for Wave 1. Furthermore, we plan to expand the study’s geographic coverage from 18 to 22 states and union territories of India. New states in Wave 2 include Andhra Pradesh, Chhattisgarh, Jharkhand, and Pondicherry. Once the data collection is complete, a longitudinal dataset and codebook will be made publicly available to the larger research community at the Gateway to Global Aging Data website (https://g2aging.org/). These longitudinal data will allow researchers to investigate changes in cognition over time and strengthen our understanding of cognitive decline and dementia incidence in India.

## Supplementary information

Supplementary Table 1

## Data Availability

Stata code used to create the Harmonized LASI-DAD dataset is available on the Gateway to Global Aging Data website at https://g2aging.org/. Users must first register to access the Gateway website. Next, users are required to sign a Data Use Agreement form to request access to the data. Once access has been approved, the user can download the Harmonized LASI-DAD data file, the code to generate the dataset, the associated codebook, and the venous blood specimen (VBS) data file.
